# Comparative analysis of videofluoroscopy and pulse oximetry for aspiration identification in patients with dysphagia after stroke and non-dysphagics

**DOI:** 10.1007/s00405-024-08613-0

**Published:** 2024-04-06

**Authors:** Serkan Bengisu, Özlem Öge-Daşdöğen, Hatice Yelda Yıldız

**Affiliations:** 1 Department of Speech and Language Therapy, Faculty of Health Sciences, Istanbul Atlas University, Istanbul, Turkey; 2 BAVIM—Stroke Center, Istinye University Liv Hospital, Istanbul, Turkey; 3 Department of Neurology, Faculty of Medicine, Istinye University, Istanbul, Turkey

**Keywords:** Aspiration, Deglutition, Dysphagia, Pulse oximetry, Swallowing, Swallowing disorders

## Abstract

**Purpose:**

Dysphagia is a prevalent symptom observed in acute stroke. Several bedside screening tests are employed for the early detection of dysphagia. Pulse oximetry emerges as a practical and supportive method to augment the existing techniques utilized during bedside swallowing assessments. Desaturation levels, as measured by pulse oximetry, are acknowledged as indicative of aspiration by certain screening tests. However, the predictive capability of pulse oximetry in determining aspiration remains a subject of controversy. The objective of this study was to compare aspiration and oxygen desaturation levels by time and aspiration severity in dysphagic patients compared to healthy controls. It also aimed to evaluate the accuracy of pulse oximetry by comparing it with VFSS findings in detecting aspiration in both liquid (IDDSI-0) and semi-solid (IDDSI-4) consistencies.

**Materials and methods:**

Eighty subjects (40 healthy and 40 acute stroke patients) participated. Patients suspected of dysphagia underwent videofluoroscopy as part of the stroke unit’s routine procedure. Baseline SpO_2_ was measured before VFSS, and stabilized values were recorded. Sequential IDDSI-0 and IDDSI-4 barium tests were conducted with 5 ml boluses. Stabilized SPO_2_ values were recorded during swallowing and 3-min post-feeding. Patients with non-dysphagia received equal bolus monitoring. Changes in SPO_2_ during, before, and after swallowing were analyzed for each consistency in both groups.

**Results:**

The study revealed a statistically significant difference in SPO_2_ between patients with dysphagia and controls for IDDSI-4 and IDSSI-0. In IDDSI-4, 20% of patients experienced SpO_2_ decrease compared to 2.5% in control group (*p* = 0.013). For IDDSI-0, 35% of patients showed SpO_2_ decrease, while none in the control group did (*p* = 0.0001). Aspiration rates were 2.5% in IDDSI-4 and 57.5% in IDDSI-0. In IDDSI-0, SpO_2_ decrease significantly correlated with aspiration (*p* = 0.0001). In IDDSI-4, 20.5% had SpO_2_ decrease without aspiration, and showing no significant difference (*p* = 0.613). Penetration-Aspiration Scale scores had no significant association with SpO_2_ decrease (*p* = 0.602). Pulse oximetry in IDDSI-4 had limited sensitivity (0%) and positive predictive value, (0%) while in IDDSI-0, it demonstrated acceptable sensitivity (60.9%) and specificity (100%) with good discrimination capability (AUC = 0.83).

**Conclusions:**

A decrease in SPO_2_ may indicate potential aspiration but is insufficient alone for detection. This study proposes pulse oximetry as a valuable complementary tool in assessing dysphagia but emphasizes that aspiration cannot be reliably predicted based solely on SpO_2_ decrease.

## Introduction

Dysphagia, a condition characterized by difficulties in swallowing, frequently occurs in acute stroke due to disruption of the normal swallowing mechanism. Dysphagia is prevalent in over half of stroke survivors [1]. Stroke is a prevalent neurological disease globally, associated with a high mortality rate [2]. In Turkey specifically, vascular system diseases are the leading cause of death, and among these diseases, cerebrovascular diseases rank as the third most common cause of mortality [3].

The presence of dysphagia may lead to various health complications such as dehydration, malnutrition, weight loss, increased hospitalization, and respiratory infections namely pneumonia, [4, 5]. Consequently, early identification and intervention in swallowing disorders are crucial to prevent the aforementioned life-threatening consequences, reduce hospital stay, and minimize long-term healthcare costs [6, 7].

National and international guidelines have recently emphasized the importance of dysphagia assessment and management in acute care settings worldwide [8–11]. Several diagnostic techniques are utilized to determine the presence of dysphagia, including bedside assessment, clinical examination, and instrumental tests. Among these techniques, the videofluoroscopic swallowing study (VFSS) is considered the gold standard for comprehensive visualization of swallowing physiology and accurate identification of aspiration [12]. However, VFSS has certain drawbacks including radiation exposure, altered food consistency and sensory feedback due to the use of contrast media, and limited availability of specialized equipment [13]. The feasibility, applicability, widespread availability, and safety of pulse oximetry for bedside assessment, complementing existing diagnostic methods were proposed in a seminal study [14].

Pulse oximetry has been shown as a potential tool in assessing dysphagia and can be utilized in conjunction with other diagnostic approaches to enhance the overall assessment process [15, 16]. Several studies have indicated that the use of pulse oximetry to monitor oxygen desaturation during and immediately after swallowing contributes to diagnosing aspiration [17–22]. Furthermore, some authors have suggested that combining bedside assessment with pulse oximeter measurements of SpO_2_ may provide valuable insights into the detection of aspiration [22]. However, some studies have indicated that pulse oximetry is not a reliable instrument to detect the risk of aspiration in dysphagia and does not support the use of pulse oximetry to detect aspiration [22–25]. It should be noted that some studies have also taken a more cautious approach to the detection of aspiration and call for further research [23].

Numerous studies on this subject have yielded different findings might be due to the different methodological factors adopted by the studies such as the definition of desaturation criteria. In the review conducted by Wang et al. [28], the potential risk of bias associated with different pulse oximetry parameters was highlighted. In their review, Britton et al. (2017) reported that among the 10 studies included in their analysis, 4 utilized a 2% SpO_2_ decrease criterion, 1 used a 3% decrease, and 1 employed a 4% decrease. Another study directly compared desaturations of 2% and 3% [25], highlighting the variability in defining desaturation criteria across these studies, with a range of 2% to 4% SpO_2_ decrease considered as a potential marker. Despite some conflicting outcomes, national guidelines still recommend a decline in SpO_2_ > 2% as a marker of aspiration [23].

The primary objective of this study was to investigate the relationship between aspiration and oxygen desaturation levels in dysphagic patients, compared to a healthy control group. To achieve this, peripheral capillary oxygen saturation (SpO_2_) is continuously monitored using pulse oximetry before, during (simultaneously with VFSS), and after swallowing considering the consistency used during VFSS. Furthermore, the study sought to determine whether there was a relationship between oxygen desaturation and the severity of aspiration. In parallel, we assessed the accuracy of pulse oximetry in detecting aspiration in both liquid and semi-solid consistencies, employing sensitivity, specificity, and area under the curve (AUC) values. By addressing these research questions, our study aimed to provide valuable insights into the potential utility of pulse oximetry as a non-invasive tool for early aspiration detection and dysphagia management.

## Materials and methods

### Participants

A sample of eighty subjects (40 healthy and 40 patients with acute stroke) participated in this study. A group of forty patients who had an acute stroke (aged 40–88 years) was compared with forty healthy subjects (aged 44–86 years). All acute stroke patients were recruited from the stroke unit of the Istinye University Liv Hospital for 12-month period in 2022. All acute stroke patients were evaluated within 24 h after the stroke by the Gugging Swallowing Screen (GUSS). Acute stroke patients suspected of having a swallowing disorder in terms of GUSS results had been referred to VFSS as a part of the routine procedure of the stroke unit. The exclusion criteria for the acute stroke patients were as follows: the inability to follow simple commands, cognitive or language impairment that precludes informed consent, receiving supplemental O_2_, having respiratory or cardiac impairments, medical history of pre-existing dysphagia, previous history of head and neck cancer, inability to sit upright position, having skin-related problems such as cutaneous disease, presence of tattoo on the index finger on the non-hemiplegic hand. Acute stroke patients were compared to 40 healthy controls recruited from the community, closely matched for age and gender with no reported dysphagia, stroke, or other neurologic impairments, respiratory and cardiac problems, and head/neck cancer history. Demographic and clinical characteristics are shown in Table 1. Each participant provided informed consent and the study was approved by the Uskudar University Ethics Committee (approval number. 61351342).

**Table 1:** Demographic and clinical characteristics of the participants

	Patient group (*n* = 40)	Healthy controls (*n* = 40)
Age, years	68.4 ± 11.9	64.05 ± 10.6
Gender (M/F)	20/20	20/20
Stroke subtype		
Ischemic stroke	38	NA
Hemorrhagic stroke	2	NA
Lesion site		
Right/left, *n* (%)	18/22 (45.0/55.0)	NA
Number of strokes, *n* (%)		
1	35 (87.5)	NA
2	4 (10)	NA
> 2	1 (2.5)	NA
NIHSS (points), mean ± SD	10.15 ± 4.9	NA

*SD* standard deviation, *NIHSS*, National Institutes of Health Stroke Scales, *NA* not applicable

### Instrumentation

The pulse oximetry was obtained by the Mindray PM-60™ Pulse Oximeter before and during the VFSS procedure. The first speech and language pathologist (SLP) monitored real-time SpO_2_ values. Philips DuoDiagnost 2008 fluoroscopy device was utilized for VFSS evaluation. Each participant was seated in a wheelchair at an upright position and was underwent VFSS to obtain lateral images of the oropharynx, larynx, and upper esophagus during modified barium swallowing.

### Procedure

Before utilizing VFSS, all patients had probes fitted to non-hemiplegic index finger which was clean. If there was nail polish, it was removed before data collection. The room was warm and dimly lighted. The participants were asked to keep their arms to which the probe was attached still. Before the initiation of the standard VFSS, baseline arterial oxygen saturation (SpO_2_) was measured for 5 min and the stabilized value was noted. Patients then underwent VFSS simultaneously using processes including liquid barium and semi-solid barium. The radiographic images were captured while applying a 5 ml amount of liquid barium and semi-solid barium tests, respectively. For all examinations, continuous fluoroscopy was employed. The videofluoroscopic swallowing studies (VFSS) were recorded and digitally captured at a rate of 30 frames per second. The participants underwent the same standardized VFSS protocol, which involved three swallowing trials (3 × tsp of 5 ml), for each of the two consistencies being tested. To provide contrast, E-Z-HD barium sulfate powder for suspension (98% *w*/*w*) was utilized as the contrast agent. In the statistical analysis, the criteria used were 5 ml IDDSI Level-0 for liquids and 5 ml IDDSI Level-4 for semi-solid consistencies, as per the guidelines set by the International Dysphagia Diet Standardization Initiative (IDDSI).

The VFSS scenes were recorded in a digital video file. The footage was evaluated using the Penetration-Aspiration Scale (PAS) and scored by another SLP blinded to the patient information, and SpO_2_ values. PAS, which operates on an 8-point clinical scale, defines the severity of penetration and aspiration and determines whether material entering the airway is expelled [26]. Moreover, before utilization of VFSS, the stabilized SPO_2_ values were recorded by the first SLP during swallowing and after the following 3-min time period of feeding to observe the effects of potentially delayed aspiration.

Non-dysphagic participants received the same amount of IDDSI-0 and IDDSI-4 bolus while being monitored on the pulse oximeter for the same period as the dysphagic patients.

### Statistical analyses

The statistical analyses were conducted using SPSS software version 26.0 (IBM SPSS Inc., Chicago, IL). The descriptive statistics for demographics including gender and age for all participants and clinical characteristics including lesion site, number of strokes, and NIHSS scores for the dysphagia group are presented in Table 1.

To assess the normality of the data distribution, the Shapiro–Wilk test was employed. For non-normally distributed data, the non-parametric Mann–Whitney *U* test was used to compare between-group differences in SpO_2_ levels of the IDDSI-0 and IDDSI-4 viscosities according to the three different time points: before, during, and after swallowing.

Additionally, a Chi-square test was conducted as part of the univariate analysis to examine the significant relationship between desaturation levels and aspiration observed during VFSS. Sensitivity, specificity, positive predictive value (PPV), negative predictive value (NPV), area under curve (AUC) and accuracy were determined presence of aspiration for swallowing trials based on IDDSI-0 and IDDSI-4 consistencies, presence of aspiration determined by VFSS and the SpO_2_ desaturation with 95% confidence intervals (95%CI). The temporal variation in oxygen saturation levels at three time points (before, during, and after swallowing) during the consumption of IDDSI-0 and IDDSI-4 consistencies was analyzed by the Friedman Test.

The significance level was set at 0.05 for interpreting the results. A *p*-value less than 0.05 indicated a statistically significant difference, while a *p*-value greater than 0.05 indicated no significant difference.

## Results

Forty patients with post-stroke dysphagia with a mean age of 68.4 ± 11.9 and 40 healthy controls with a mean age of 64.05 ± 10.6, a total of 80 participants, took part in the study. Twenty female (50%) and 20 male (50%) participants took part in both participant groups. Regarding the stroke subtypes, 38 (95%) were diagnosed with ischemic stroke and 2 (5%) had a hemorrhagic stroke. As for the lesion site, 18 (45%) and 22 (55%) had lesions in the right and left hemispheres, respectively. Thirty-five patients (35%) had a stroke for the first time, four patients (10%) had a stroke for the second time and one patient (2.5%) had a stroke for more than two strokes. The mean stroke severity of the patient group was 10.15 ± 4.9 according to the National Institute of Health Stroke Scale (NIHSS).

The data in Table 2 indicate that there were significant differences among the two groups on SpO_2_ values in terms of different time points including during and after the swallowing in both consistencies.

**Table 2:** SpO_2_ values before, during, and after swallowing trials using two different consistencies both for patient and healthy control group

	Patient group Mean ± SD (*n* = 40)	Healthy control group Mean ± SD (*n* = 40)	*U*	*Z*	*p*
IDDSI Level-4					
SpO_2_ before swallowing	96.25 ± 1.9	97.03 ± 1.3	601	− 1.957	0.051
SpO_2_ during swallowing	95.8 ± 2.0	96.95 ± 1.5	521	− 2.728	0.006**
SpO_2_ after swallowing	95.7 ± 1.9	96.93 ± 1.5	481.5	− 3.113	0.002**
IDDSI Level-0					
SpO_2_ before swallowing	96.23 ± 1.8	96.95 ± 1.4	601	− 1.947	0.052
SpO_2_ during swallowing	95.45 ± 2.4	97 ± 1.3	465	− 3.724	0.001**
SpO_2_ after swallowing	95.43 ± 2.1	97.23 ± 1.4	382	− 4.086	0.0001**

Bold values indicate statistically significant values

*p < 0.05

**p < 0.01 is considered significant

A statistically significant difference was obtained between the oxygen saturation values obtained during and after swallowing in both IDDSI-4 and IDDSI-0 consistency between the patient group and the healthy control group (*p* = 0.006, *p* = 0.002, *p* = 0.001, *p* = 0.0001 < 0.01).

It was examined whether there was a difference between the patient group and the healthy control group in terms of a decrease of ≥ 2% in SpO_2_ values and the results obtained are shown in Table 3.

**Table 3:** SpO_2_ changes according to consistency between patient and healthy control group

IDDSI Level-4	SpO_2_ ≥ 2% drop		χ^2^	*p*
	None	Yes		
Patient group	32 (80%)	8 (20%)	6.135	0.013*
Healthy control group	39 (97.5%)	1 (2.5%)		

IDDSI Level-0	None	Yes		
Patient group	26 (65%)	14 (35%)	16.97	0.0001**
Healthy control group	40 (100%)	0 (0%)		

Bold values indicate statistically significant values

*p < 0.05

**p < 0.01 is considered significant

A decrease in SpO_2_ value was observed in 8 (20%) patients in the patient group in IDDSI-4 consistency, while a decrease in SpO_2_ value was observed in only 1 (2.5%) patient in the control group. There was a statistically significant difference between the groups in terms of a decrease in saturation ≥ 2% in IDDSI-4 consistency (*p* = 0.013). In IDDSI-0 consistency, a decrease in SpO_2_ value was observed in 14 (35%) patients, and no decrease in SpO_2_ value was observed in any participant in the control group. There was a statistically significant difference between the groups in terms of a decrease in saturation ≥ 2% in IDDSI-0 consistency (*p* = 0.0001).

Whether there was a change in oxygen saturation values in the patient group at two different time points according to the presence of aspiration was analyzed descriptively and the results obtained are shown in Table 4.

**Table 4:** Oxygen saturation values according to the presence of aspiration before and after swallowing trials in the patient group

	IDDSI Level-4	IDDSI Level-0
Aspiration	1 (2.5%)	23 (57.5%)
Before swallowing SpO_2_ values ± SD	NA	96.04 ± 2
After swallowing SpO_2_ values ± SD	NA	94.61 ± 2
Decrease in SpO_2_ ≥ 2%, *n* (%)	0 (0%)	14 (60.9%)
No aspiration	39 (97.5%)	17 (42.5%)
Before swallowing SpO_2_ values ± SD	96.31 ± 1.9	96.47 ± 1.5
After swallowing SpO_2_ values ± SD	95.74 ± 1.9	96.53 ± 1.7
Decrease in SpO_2_ ≥ 2%, *n* (%)	8 (20.5%)	0 (0%)

*NA* not applicable

In IDDSI-4 consistency, aspiration was not observed in 39 (97.5%) of 40 patients, while aspiration was observed in 1 (2.5%) patient. However, no decrease in SpO_2_ value was observed in the patient with aspiration. A decrease in SpO_2_ value was observed in 8 (20.5%) of 40 patients without aspiration. Before swallowing, the mean SpO_2_ level of the patients was 96.31 ± 1.9, whose SpO_2_ value was already decreased without aspiration and the mean SpO_2_ level after swallowing was 95.74 ± 1.9.

In IDDSI-0 consistency, aspiration was observed in 23 (57.5%) of 40 patients, while aspiration was not observed in 17 (42.5%) patients. In 14 (60.9%) of 23 patients with aspiration, a decrease of ≥ 2% in SpO_2_ value was found. The mean SpO_2_ level of aspirating patients before swallowing was 96.04 ± 2 and the mean SpO_2_ level after swallowing was 94.61 ± 2. In none of the 17 patients in whom aspiration was not observed in IDDSI Level-0 consistency, a decrease of > 2% in SpO_2_ value was detected. The mean SpO_2_ level of the patients without aspiration was 96.47 ± 1.5 before swallowing and 96.53 ± 1.7 after swallowing.

It was analyzed whether there was a relationship between a decrease of ≥ 2% in SpO_2_ value seen in measurements and aspiration detected with VFSS. The results obtained are shown in Table 5.

**Table 5:** Relationship between SpO_2_ desaturation and presence of aspiration documented by VFSS in dysphagic patients

	Decrease in SpO_2_ ≥ 2%		*χ*^2^	*p*
	None	Yes		
IDDSI Level-4				
No aspiration	31 (79.5%)	8 (20.5%)	0.256	0.613
Aspiration	1 (100%)	0 (0%)		
IDDSI Level-0				
No aspiration	17 (100%)	0 (0%)	15.92	0.0001**
Aspiration	9 (39.1%)	14 (60.9%)		

Bold values indicate statistically significant values

*p < 0.05

**p < 0.01 is considered significant

Comparing the results of pulse oximetry and the VFSS findings in IDDSI-0 consistency, 14 patients (60.9%) out of 23 patients who had an aspiration finding showed significant oxygen desaturation as well (SpO_2_ drops exceed 2%). Of 17 patients with no aspiration on VFSS, (100%) none had a decrease in SpO_2_ of more than ≥ 2%. There was a significant relationship between a decrease in SpO_2_ and aspiration on VFSS examination using the Chi-square test (*p* = 0.0001).

In IDDSI-4 consistency, among 39 patients without aspiration, 31 (79.5%) exhibited no oxygen desaturation, while 8 (20.5%) experienced a ≥ 2% decrease in SpO_2_ without aspiration. Notably, oxygen desaturation was not observed in one patient with aspiration at this consistency. There was no significant difference between a decrease in SpO_2_ and aspiration on VFSS in IDSSI-4 consistency (*p* = 0.613).

The sensitivity and specificity, predictive values, and area under curve (AUC) of pulse oximetry in each consistency was evaluated by VFSS compared in 40 patients and the results obtained are shown in Table 6.

**Table 6:** Analysis of SpO_2_ desaturation and presence of aspiration on IDSSI-4 and IDDSI-0 consistencies on VFSS. **AUC,** area under curve

Consistency	Sensitivity (95% CI)	Specificity (95% CI)	Positive predictive value (95% CI)	Negative predictive value (95% CI)	AUC
IDDSI Level-4	0.0%	79.5% (63.5–90.7%)	0.0%	96.9% (96.4–97.3%)	0.48 (0.26–0.71)
IDDSI Level-0	60.9% (38.5–80.3%)	100% (80.5–100.0%)	60.9% (53.2–75.8%)	100% (93.5–100%)	0.83 (0.70–0.95)

**Fig. 1:**
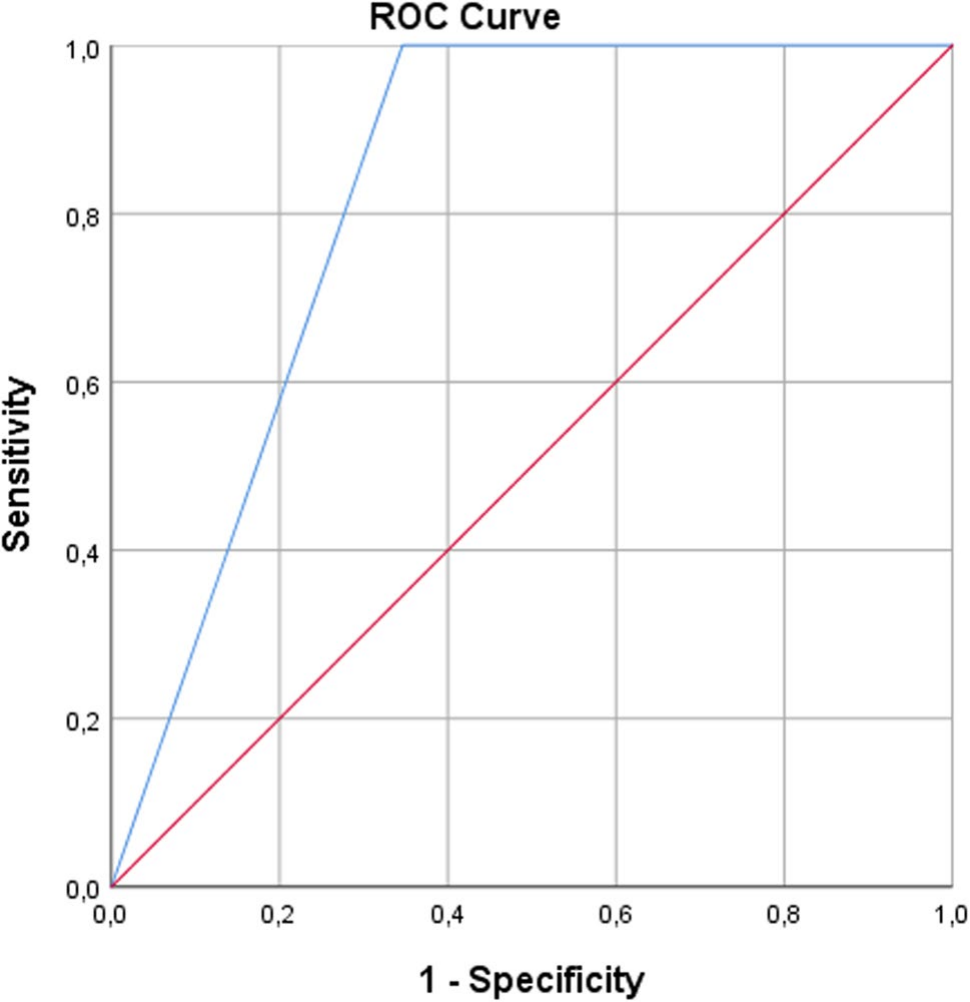
Receiver operating curve (ROC) analysis of SpO_2_ desaturation and presence of aspiration on IDDSI-0 consistencies on VFSS

For IDDSI-4 consistency, the SpO_2_ desaturation in aspiration had a sensitivity of 0%, specificity of 79.5% (95%CI 63.5–90.7%), PPV of 0%, NPV of 96.9% (95%CI 96.4–97.3%) and AUC of 0.48. For IDDSI-0 consistency, the SpO_2_ desaturation in aspiration had a sensitivity of 60.9% (95%CI 38.5–80.3%), specificity of 100%(95%CI 80.5–100.0%), PPV of 60.9% (95%CI 53.2–75.8%), NPV of 100% (95%CI 93.5–100%) and AUC of 0.83 (Fig. 1).

**Fig. 2:**
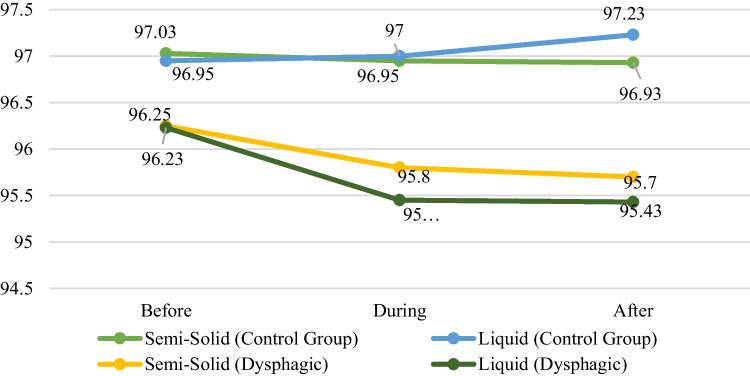
Changes in SpO_2_ values in IDDSI-4 and IDDSI-0 consistencies of patient and control group before, during, and after swallowing

The temporal changes in SpO_2_ values before, during, and after swallowing in the IDDSI-4, and IDDSI-0 consistencies were examined between the patient and control groups, and the results obtained are presented in Fig. 2.

There was a statistically significant difference in SpO_2_ values before, during, and after swallowing in the dysphagic group for both IDDSI-4 and IDDSI-0 consistencies (*p* = 0.007, *p* = 0.003). The difference was attributed to the variations between SpO_2_ values before and during swallowing, as well as between SpO_2_ values before and after swallowing.

In the healthy control group, there was no statistically significant difference in SpO_2_ values before, during, and after swallowing for the IDDSI-4 and IDDSI-0 consistency (*p* = 0.469, *p* = 0.521).

The relationship between the score obtained from the Penetration-Aspiration Scale (PAS) and the observed decrease in SpO_2_ values in patients with aspiration was examined, and the results are shown in Table 7.

**Table 7:** Oxygen saturation values before, during and after swallowing in dysphagics with aspiration based on PAS scores

VFSS Result	Baseline SpO_2_ x̄ ± SD	During SpO_2_ x̄ ± SD	After SpO_2_ x̄ ± SD	Range of SpO_2_ decline	*p*
PAS 6 (*n* = 3)	96.67 ± 1.5	94.67 ± 1.5	94.67 ± 1.5	2.0	
PAS 7 (*n* = 14)	95.57 ± 1.8	94.57 ± 1.4	94.29 ± 1.2	1.3	0.602
PAS 8 (*n* = 6)	96.83 ± 2.6	95.17 ± 2.9	95.33 ± 2.7	1.5	

*VFSS* videofluoroscopy, *PAS* Penetration-Aspiration Scale, *PAS 6* aspirated and cleared, *PAS 7* aspirated and not cleared, *PAS 8* silent aspirated

Based on the VFSS results, it was found that three dysphagic patients demonstrated a PAS score of 6, while 14 dysphagic patients demonstrated a PAS score of 7, and six dysphagic patients were observed to have a PAS score of 8. In IDDSI-4 consistency, aspiration was seen in only one patient out of 40 and the PAS score was 7. In IDDSI-0 consistency, aspiration was observed in 23 (57.5%) out of 40 patients according to PAS scores. There was no statistically significant difference in saturation changes between different PAS scores including PAS 6 (aspirated and cleared), PAS 7 (aspirated and not cleared), and PAS 8 (silent aspirated) (*p* = 0.602).

## Discussion

In the present study, efforts were made to characterize in detail the relationship between oxygen desaturation and the presence of aspiration. For this purpose, pulse oximetry was utilized in patients with dysphagia before, during (simultaneously applying VFSS), and after-swallowing in the acute post-stroke period and compared with pulse oximetry values of individuals without dysphagia. In addition, it was determined whether there was a relationship between the presence of aspiration and oxygen desaturation according to consistency. It was also investigated whether there was a relationship between oxygen desaturation according the severity of aspiration determined by PAS.

In our study, aspiration was observed during VFSS evaluation in approximately 60% of acute stroke patients with suspected dysphagia according to GUSS, oxygen desaturation was detected simultaneously in more than half of them. The finding is consistent with studies supporting a decrease in SPO_2_ values during and/or after swallowing during aspiration [17–19, 21] but contradicts the findings of studies reporting that SPO_2_ may not be a reliable indicator of aspiration [20, 22, 27, 28]. As aforementioned, several studies support that there is an association between aspiration and oxygen desaturation. Measurement tools used to determine dysphagia and aspiration, such as the Volume-Viscosity Swallowing Test [29], included pulse oximetry to detect silent aspiration, which occurs in about half of the patients. They concluded that pulse oximetry increases the diagnostic sensitivity of this test. However, the review by Britton et al. concluded that those inconsistent findings may be due to methodological differences or even different definitions of desaturation, and therefore the available evidence is not sufficient to support the use of pulse oximetry in detecting aspiration [25]. One reason for the inconsistent findings might be the clinically recognized phenomenon that aspiration does not necessarily occur with each swallow [30].

Despite these challenges, accurately defining silent aspiration and penetration accurately with only screening tests remains difficult. However, the Volume-Viscosity Swallowing Test (V-VST) asserts that by integrating pulse oximetry as a complementary measure, it provides a more reliable evaluation of dysphagia. Recent findings from the V-VST study demonstrate promising results, with 83.7% sensitivity and 64.7% specificity for bolus penetration into the larynx, and 100% sensitivity and 28.8% specificity for aspiration. These findings suggest that incorporating diagnostic accuracy and provide clinicians with more robust tools for assessing dysphagia and aspiration [29, 31].

In terms of consistency, in participants who had no complaints about swallowing and were assigned to the healthy control group, none of the participants had a decrease in SPO_2_ at IDDSI-0 consistency, whereas in the dysphagia group, 35% of patients had a decrease in SPO_2_. Aspiration was detected in approximately 60% of all patients in the IDDSI-0 consistency. It was determined that aspiration was accompanied by a decrease in SPO_2_ in approximately 60% of patients with aspiration. In IDDSI-4 consistency, oxygen desaturation was detected in only one participant in the non-dysphagic group, while oxygen desaturation was detected in approximately 1/5 of the dysphagic group despite the absence of aspiration, and no decrease in SPO_2_ was detected in only one participant with dysphagia despite aspiration. The data obtained indicate that there is a relationship between oxygen desaturation and aspiration depending on the consistency. When examined according to consistency, no significant difference was observed in oxygen saturation according to consistency in those without dysphagia, while oxygen desaturation decreased more in IDDSI-0 consistency than in IDDSI-4 consistency in the dysphagia group. The mechanism of oxygen desaturation during dysphagia is different from the normal swallowing process. Most likely, the bolus entering the larynx narrows the airway by stimulating the chemical receptors in the larynx and it causes a decrease in SPO_2_ [20, 32]. Therefore, aspiration of a solid bolus might be more likely to result in airway occlusion. However, some researchers theoretically argue that a decrease in SPO_2_ due to the presence of a bolus at the laryngeal level does not make sense, as airway obstruction would occur at the level of the lower respiratory system or lungs [25]. In our study, the frequency of aspiration with a decrease in SPO_2_ and the accompaniment of aspiration with a decrease in SPO_2_ displayed different patterns in IDDSI-0 and IDDSI-4 consistencies. Thin liquids such as water are known to pose a safety issue for people with dysphagia due to their rapid flow [33, 34]. In line with this, we found more aspiration in IDDSI-0 than in IDDSI-4 consistency, and more than half of those were accompanied by oxygen desaturation. Nonetheless, it should be noted that despite the absence of aspiration, oxygen desaturation might still be observed with IDDSI-4 consistency. Conversely, there might not be observed oxygen desaturation despite the presence of aspiration. This discrepancy suggests a decrease in the sensitivity of pulse oximetry especially when assessing semi-solid consistency. This finding suggests that the compatibility of aspiration and desaturation varies depending on the consistency. Butler et al. also indicated that the aspiration risk may vary according to consistency [35].

The sensitivity rate for IDDSI-4 consistency indicates that pulse oximetry did not exhibit the capacity to correctly identify true positive cases. The lack of sensitivity implies that pulse oximetry failed to detect dysphagia in IDDSI-4 consistency. However, the specificity value in IDDSI-4 consistency indicates the pulse oximetry’s ability to accurately true negative cases, implying its proficiency in identifying non-dysphagics. The low PPV further suggests that pulse oximetry was unable to reliably predict true positive cases, while the high NPV indicates that pulse oximetry is quite accurate in correctly identifying individuals without dysphagia. The AUC value suggests a limited ability of the pulse oximetry to distinguish between positive and negative cases. The findings raise concerns about the effectiveness of pulse oximetry in accurately diagnosing individuals with dysphagia in semi-solid consistency.

The good sensitivity rate in IDDSI-0 consistency indicates that pulse oximetry can effectively capture a substantial portion of true positive cases. Moreover, the high specificity in IDDSI-0 consistency underscores the pulse oximetry’s ability to accurately identify true negative cases. Furthermore, the AUC value suggests that pulse oximetry has good discriminatory power in IDDSI-0 consistency, allowing it to effectively distinguish between cases with and without dysphagia. The combination of sensitivity, specificity, and NPV, along with the AUC value within the context of IDDSI-0 consistency, suggests a potential diagnostic capability of pulse oximetry in detecting aspiration during liquid swallowing.

Our study measured oxygen desaturation by finger pulse oximetry at three different time points: before, during, and after swallowing. We found no difference between the patient and healthy control group in SPO_2_ values before-swallowing in both consistencies, but we found a significant difference at the time points including during- and after-swallowing. However, while this difference was statistically significant, the difference between the SPO_2_ values measured at the time points before, during and after swallowing in the patient group was less than 1%.

One of the crucial mechanisms to protect the airway is the coordination between respiration and swallowing [36]. If the temporal coupling between respiration and swallowing is disrupted, it can cause the prolongation of swallowing latency leading to a delay in the timing of the swallowing. Patients with dysphagia may be more likely to develop aspiration pneumonia if there is a higher degree of discoordination between their respiration and swallowing cycle [37].

Park et al. indicated that stroke patients with dysphagia had a significantly longer delay in the laryngeal closure [38]. The fact that the time point after-swallowing was statistically more significant in terms of SPO_2_ values than the time point during-swallowing might be due to the temporal delay in the detection of oxygen desaturation in finger pulse oximetry due to airway obstruction. In contrast, Huang et al. found that laryngeal closure duration is shorter in post-stroke dysphagic patients than the healthy controls and the delay increases the risk of penetration and aspiration [39]. When the time points were analyzed according to the consistencies, a statistically significant difference was found between dysphagic patients and healthy controls in SPO_2_ values both after- and during-swallowing in IDDSI-0 consistency compared to IDDSI-4 consistency, respectively. Hiss et al. found differences in the timing of the onset of respiratory pause because of bolus consistency [40]. The authors explained this finding as being related to the flow rate of the consistencies and that physiological events, including the onset of respiratory pause, may occur more slowly in thick consistencies than in thin consistencies. In our study, the statistical difference in the time points in IDDSI-0 consistency compared to the time points in IDDSI-4 consistency may be due to the flow rate of the consistencies. However, there is an ongoing debate regarding the impact of bolus on the timing and duration of respiratory pauses [41].

We endeavor to control the potential confounding factors mentioned in the literature on whether oxygen desaturation can be used to determine dysphagia. In our study, we limited the age range and included patients with the same disease etiology such as stroke, and compared the data obtained with a comparison group of normal healthy subjects. We determined the effect of variables such as both aspiration severity and food consistency on oxygen desaturation. We used a standardized method such as IDDSI to determine food consistency. In addition, we used VFSS and PAS during swallowing and collected the data blindly. To prevent artifacts, we controlled parameters such as room temperature, the presence of nail polish, the presence of a tattoo, keeping the arm still to which, the oximeter was connected, cleaning the finger, and attaching the probe to the finger on the non-hemiplegic side. One of our limitations is that we could not assess the pulmonary function of dysphagic patients before data collection, although we excluded patients with respiratory or cardiac functions. Another limitation is that we relied on the participants' self-report of whether they had dysphagia while assigning them to the healthy control group. One of the limitations of this study, we did not include non-dysphagic post-stroke patients. Comparing three groups could have more comprehensive understanding of the discriminative ability, reliability, and specificity, and it could provide insights into the generalizability of the findings. The other limitation of our study was the inadequate number of dysphagic patients assigned all three different Penetration-Aspiration Scale (PAS) levels. Although we did not observe a statistically significant decline in oxygen saturation levels before, during, and after swallowing across the three PAS levels, we acknowledge the need for future studies to evaluate comprehensively SPO_2_ levels in groups with varying aspiration severity based on PAS ratings. Particularly in silent aspiration, a decline in SPO_2_ might be a crucial marker. Therefore, we might expect a higher decrease in SPO_2_ in PAS 8. However, when the mean values of the SPO_2_ levels of the patients in the PAS 8 group were analyzed; the decrease between before and after time points was found to be 1.5% and remained below the 2% change. Despite the limited number of patients with aspiration, the present results indicate that pulse oximetry alone may not be sufficiently effective in detecting aspiration.

Furthermore, we believe that examining SPO_2_ levels concerning aspiration awareness levels would provide valuable insights. Additionally, incorporating a multimodal sensory assessment to identify the presence of hypesthesia and exploring its association with aspiration severity and the degree of SPO_2_ decline might further enhance our understanding of the field.

In further studies, more reliable results can be obtained by using measurement of multimodal physiological signals with devices such as nasal airflow and nasal temperature sensors, plethysmography, electromyography, electrocardiogram, and skin conductance meter in addition to pulse oximetry for monitoring swallowing and respiratory cycle to detect dysphagia. In addition, complementary screening techniques such as measurement of laryngeal elevation and/or cervical auscultation may be included in the assessment process. These methods offer the advantages of being relatively low-cost and easily accessible.

The findings of this study highlight the importance of considering the consistency of ingested materials when utilizing pulse oximetry as a diagnostic tool for detecting aspiration in dysphagic patients. Further research and evaluation of pulse oximetry’s performance in larger patient populations are needed to validate its effectiveness in aspiration detection for both consistencies. Additionally, it is recommended to consider SPO_2_ decrease exceeding 2% as a criterion for improved accuracy.

A decrease in SPO_2_ may hold the potential to predict aspiration, contingent upon the consistency being considered. However, detecting aspiration alone is not sufficient. This study suggested that pulse oximetry could serve complementary tool in assessing individuals with dysphagia. However, aspiration occurring on VFSS cannot be predicted based on decrease in SpO_2_ in pulse oximetry.

Taken together, oxygen saturation monitorization during and after prandial swallowing may have the potential to improve complementary assessments such as bedside swallowing assessment and provide a more comprehensive approach to aspiration detection. However, further research is needed to confirm these findings.

## Data Availability

The data used to support the findings of this study are included within the article.
